# Pancreatic Agenesis due to Compound Heterozygosity for a Novel Enhancer and Truncating Mutation in the PTF1A Gene

**DOI:** 10.4274/jcrpe.4494

**Published:** 2017-09-01

**Authors:** Monica Gabbay, Sian Ellard, Elisa De Franco, Regina S. Moisés

**Affiliations:** 1 Federal University of São Paulo, Paulista School of Medicine, Division of Endocrinology, São Paulo, Brazil; 2 University of Exeter Medical School, Institute of Biomedical and Clinical Science, Exeter, United Kingdom

**Keywords:** Pancreatic agenesis, neonatal diabetes, PTF1A gene

## Abstract

Neonatal diabetes, defined as the onset of diabetes within the first six months of life, is very rarely caused by pancreatic agenesis. Homozygous truncating mutations in the PTF1A gene, which encodes a transcriptional factor, have been reported in patients with pancreatic and cerebellar agenesis, whilst mutations located in a distal pancreatic-specific enhancer cause isolated pancreatic agenesis. We report an infant, born to healthy non-consanguineous parents, with neonatal diabetes due to pancreatic agenesis. Initial genetic investigation included sequencing of KCNJ11, ABCC8 and INS genes, but no mutations were found. Following this, 22 neonatal diabetes associated genes were analyzed by a next generation sequencing assay. We found compound heterozygous mutations in the PTF1A gene: A frameshift mutation in exon 1 (c.437_462 del, p.Ala146Glyfs*116) and a mutation affecting a highly conserved nucleotide within the distal pancreatic enhancer (g.23508442A>G). Both mutations were confirmed by Sanger sequencing. Isolated pancreatic agenesis resulting from compound heterozygosity for truncating and enhancer mutations in the PTF1A gene has not been previously reported. This report broadens the spectrum of mutations causing pancreatic agenesis.

**What is already known on this topic?**

Homozygous truncating mutations in PTF1A have been reported in patients with pancreatic and cerebellar agenesis, while recessive mutations located in a distal PTF1A enhancer cause isolated pancreatic agenesis.

**What this study adds?**

This is the first report of a patient with isolated pancreatic agenesis resulting from compound heterozygosity for truncating and enhancer mutations in the PTF1A gene. This study broadens the spectrum of mutations causing pancreatic agenesis and the phenotypic variability of this condition.

## INTRODUCTION

Neonatal diabetes, defined as onset of diabetes within the first 6 months of life, is a genetically heterogeneous condition with 22 known genetic causes (1,2,3). Its causal genes are involved in the development of the pancreas or islets; beta cell apoptosis or destruction; or beta-cell function (1). The PTF1A gene on chromosome 10 encodes a transcription factor with a key role in early pancreas development and cerebellar neurogenesis (4,5). Homozygous truncating mutations in PTF1A have been reported in patients with pancreatic and cerebellar agenesis, whilst mutations located in a distal pancreatic-specific enhancer cause isolated pancreatic agenesis (5,6,7,8).

Here, we report a patient with isolated pancreatic agenesis due to compound heterozygous mutations in PTF1A: A coding frameshift mutation (p.Ala146Glyfs*116) and a novel regulatory mutation located in the distal enhancer, 25 kb downstream of this gene.

## CASE REPORT

**Figure 1:**
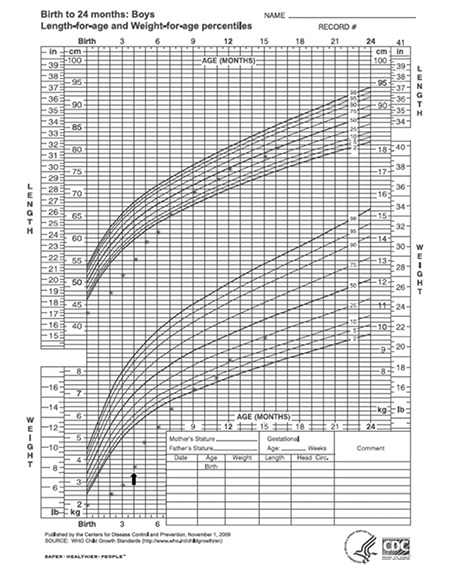
Growth of the patient plotted on the World Health Organization growth chart. Arrow denotes the start of pancreatic enzyme replacement

The index patient is a 1-year and 9-month-old boy, the second child of healthy, Caucasian, non-consanguineous parents. He was born with a birth weight of 1,935 g (below the third percentile), length of 43 cm (below the third percentile), and head circumference of 32 cm (third percentile). Intrauterine growth retardation was noted at 34 weeks of gestation, and the delivery was by cesarean section at 37 weeks of gestation. Physical examination revealed no dysmorphic features. During the first week of life, hyperglycemia (blood glucose=250 mg/dL) without ketoacidosis was observed, and treatment with subcutaneous NPH insulin was initiated on the 7^th^ day of life, followed by continuous subcutaneous insulin infusion. Despite intensive insulin therapy, the infant did not show satisfactory weight gain and at 4 months of age, his weight was 3,670 g (still below the third percentile). Abdominal distension and fatty stools were noted. Abdominal ultrasound was performed at that time and the pancreas could not be identified. No other abnormality was found. Pancreatic enzyme replacement was begun at the age of 4 months and a catch-up growth occurred (Figure 1). Over the subsequent months, his growth was satisfactory and his developmental milestones were all reached at an appropriate age.

### Genetic Analysis

**Figure 2:**
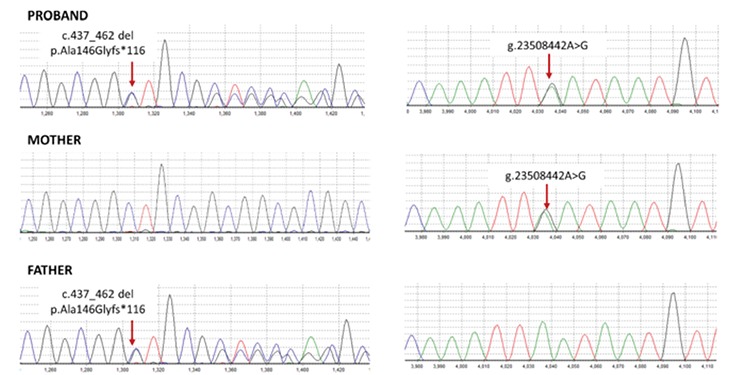
Sequence chromatograms showing the PTF1A mutations identified in the proband and his parents

Initial Sanger sequencing of the coding and flaking intronic regions of the KCNJ11 (NM_000525), ABCC8 (NM_000352), and INS (NM_000207) genes was undertaken as previously described (3), but no mutations were found. Following this, a next-generation sequencing assay was performed to analyze the coding regions and conserved splice sites of the 22 neonatal diabetes genes: KCNJ11, ABCC8, INS, EIF2AK3, FOXP3, GATA4, GATA6, GCK, GLIS3, HNF1B, IER3IP1, PDX1, PTF1A, NEUROD1, NEUROG3, NKX2-2, RFX6, SLC2A2, SLC19A2, STAT3, WFS1, and ZFP57 (Agilent custom capture v5.1/Illumina HiSeq) at the Molecular Genetics Laboratory, University of Exeter Medical School, UK (9). A compound heterozygous mutations in the PTF1A gene was found: a frameshift mutation in exon 1 (c.437_462 del, p.Ala146Glyfs*116) and a mutation affecting a highly conserved nucleotide within the distal pancreatic enhancer (g.23508442A>G) (Figure 2). The two mutations were predicted to be pathogenic and likely to be pathogenic, respectively, according to the American College of Medical Genetics and Genomics variant interpretation guidelines (10). Both mutations were confirmed by Sanger sequencing. Family member testing showed that the proband’s mother is a heterozygous carrier of the distal enhancer mutation and the father is a heterozygous carrier of the frameshift mutation.

## DISCUSSION

Pancreatic agenesis characterized by exocrine pancreatic insufficiency and permanent neonatal diabetes (11) is a rare condition. An international cohort study of patients with diabetes diagnosed before 6 months of age found that only 4.9% of them had pancreatic agenesis (12). Mutations in genes that encode transcription factors with a key role in pancreatic development, such as PDX1, GATA6, GATA4, and PTF1A, have been reported as genetic causes of congenital absence of the pancreas (5,6,7,8,12,13,14). However, the origin of this disorder remains unknown in ~15% of patients (De Franco E, unpublished data). Here, we report a patient with neonatal diabetes and exocrine pancreas insufficiency resulting from compound heterozygous mutations in the PTF1A gene. PTF1A, a 48kDa binding subunit of the pancreatic transcription factor 1 (PTF1), is required for normal pancreas development (4). This transcription factor is also expressed in embryonic neural tissues and plays a role in cerebellar neurogenesis. Ptf1a^-/-^ mice show a size reduction in the cerebellar primordium in embryos, resulting in cerebellar agenesis at birth (5). Consistently with this, recessive loss of function mutations in the PTF1A gene have been previously reported to cause agenesis of the pancreas and the cerebellum with additional dysmorphic features (5,6,15). A homozygous missense mutation, p.Pro191Thr, resulting in a protein with a 75% reduced transactivation activity, has been recently reported in patients with isolated pancreatic aplasia/hypoplasia, indicating a correlation between coding mutation severity and phenotype (16). Furthermore, mutations in the enhancer region located 25 kb downstream from the coding region of the PTF1A gene, which acts as a developmental enhancer of this gene, have been found to cause isolated pancreatic hypoplasia/agenesis, sparing the cerebellum (7,8).

To the best of our knowledge, this is the first report of a patient with isolated pancreatic agenesis resulting from compound heterozygosity for truncating and enhancer mutations in the PTF1A gene. Regarding the age of onset of diabetes, previous reports of patients with PTF1A truncating mutations showed that they had diabetes in the first month of life (5,6,15). However, patients with PTF1A enhancer mutations had phenotypic variability: The majority of cases are diagnosed in the first month of life, but diabetes at later ages was also observed (8). The patient we report had diabetes diagnosed in the first week of life and his neurological development has been normal, indicating no associated anomaly in the cerebellum. Interestingly, the lack of a severe neurological phenotype in patients with homozygous/compound heterozygous regulatory mutations has been recently reported for another congenital disease, polycystic kidney disease with hyperinsulinemic hypoglycemia (HIPKD) (17). A specific promoter mutation in the PMM2 gene, either homozygous or in trans with a coding PMM2 mutation, was reported to cause HIPKD in 11 families. Homozygous coding mutations in PMM2 have been previously reported to cause a congenital disorder of glycosylation type 1a, a severe multisystem disease with prominent neurologic features which were not observed in patients with the promoter mutation. The phenotype-genotype relationship observed in patients with coding versus non-coding mutations in PTF1A and PMM2 highlights the fundamental role of non-coding sequences in development of specific organs.

In summary, we report the case of a patient with isolated pancreatic agenesis due to compound heterozygosity for a truncating and novel enhancer mutation in PTF1A, broadening the spectrum of mutations causing pancreatic agenesis and phenotypic variability of this condition.
